# Lymph Node Immune Profiles as Predictive Biomarkers for Immune Checkpoint Inhibitor Response

**DOI:** 10.3389/fmolb.2021.674558

**Published:** 2021-05-24

**Authors:** Emily F. Goode, Evanthia T. Roussos Torres, Sheeba Irshad

**Affiliations:** ^1^ Institute of Cancer Research, London, United Kingdom; ^2^ Cancer Research UK (CRUK) Clinical Fellow, London, United Kingdom; ^3^ Norris Comprehensive Cancer Center, Keck School of Medicine, University of Southern California, Los Angeles, CA, United States; ^4^ School of Cancer and Pharmaceutical Sciences, King's College London, London, United Kingdom; ^5^ Cancer Research UK (CRUK) Clinician Scientist, London, United Kingdom

**Keywords:** lymph node, immune checkpoint inhibitor (ICI), biomarker, immune microenviroment, immune profiling, immune surveillance

## Abstract

The need for predictive biomarkers that can accurately predict patients who will respond to immune checkpoint inhibitor (ICI) immunotherapies remains a clinically unmet need. The majority of research efforts have focused on expression of immune-related markers on the tumour and its associated tumour microenvironment (TME). However, immune response to tumour neoantigens starts at the regional lymph nodes, where antigen presentation takes place and is regulated by multiple cell types and mechanisms. Knowledge of the immunological responses in bystander lymphoid organs following ICI therapies and their association with changes in the TME, could prove to be a valuable component in understanding the treatment response to these agents. Here, we review the emerging data on assessment of immunological responses within regional lymph nodes as predictive biomarkers for immunotherapies.

## Introduction

Immunotherapy, such as immune checkpoint inhibition (ICI), is now used to treat several types of cancer including melanoma, renal cell carcinoma (RCC), non-small cell lung cancer (NSCLC), head and neck cancers and some triple negative breast cancers (Borghaei et al., 2015; Larkin et al., 2015; Larkin et al., 2015; Le et al., 2015; Seiwert et al., 2016; Balar et al., 2017; Motzer et al., 2018; Schmid et al., 2020). The most frequently used immunotherapy drugs include monoclonal antibodies versus immune checkpoints such as programmed cell death–1 (PD-1), its ligand (PD-L1) and cytotoxic T lymphocyte–associated protein 4 (CTLA-4). Response rates vary between 15–53% depending on tumour type and, for those that do respond, it holds the potential to induce durable clinical benefit. However, despite the breakthrough in clinical treatment with ICIs, a proportion of patients do not respond and for others treatment duration is limited due to immune related adverse events (irAEs) which can affect any organ. Immunotherapy with anti-PD-1 or anti-CTLA-4 therapy has been associated with significant (grade 3 or 4) toxicity in 10–26% (Hodi et al., 2010; Larkin et al., 2015; Robert et al., 2015; Reck et al., 2016; Weber et al., 2017) of patients receiving monotherapy and up to 55% (Larkin et al., 2015) of those receiving combination regimens. Beyond the toxicity reported in clinical trial follow up, there is concern that late onset toxicity may occur in long-term follow up of survivors, including cardiac toxicity (Tocchetti et al., 2019). Recent progress to identify patients predisposed to irAEs, and their mechanism, has been reviewed extensively elsewhere (Mangan et al., 2020).

**FIGURE 1:**
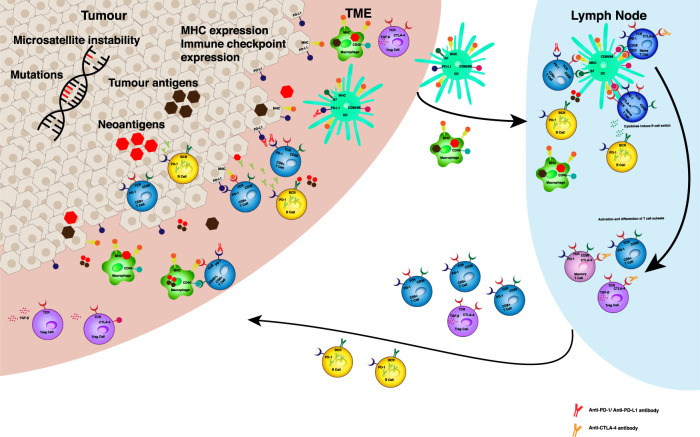
Predictive biomarkers for ICB efficacy. Tumour intrinsic factors such as genetic instability and mutational load can increase the production of tumour neoantigens in the TME. Antigen is taken up by APCs in the TME. MHC expression on tumour cells, macrophages and dendritic cells can effect antigen presentation to resident T cells and in local lymph nodes. The presence of upregulated immune checkpoints, such as PD‐1, PD‐L1, CTLA‐4 can result in inhibitory signalling to prevent effective activation and differentiation of immune cell susbsets in the TME and lymph node. Levels of expression of these receptors and their ligands may determine the impact of respective monoclonal antibody treatments on anti‐tumour responses. The balance of T cell subsets and B cells determines the immune suppressed or activated status of the anti‐tumour immune response. Anti‐PD‐1 antibodies act in the tumour microenvironment (TME) by disrupting the negative regulation of anti-tumour immunity mediated by PD-1 and its ligands (PD‐L1 and PD‐L2) expressed on somatic cell types and antigen presenting cells (APCs) respectively. Upon exposure to tumour antigen, tumour infiltrating lymphocytes express PD-1 and release pro-inflammatory cytokines, such as interferon‐ γ, which trigger PD‐L1 expression in cells in the TME. PD‐1/PD‐L1 interaction results in T cell exhaustion and inhibition of the antitumour cytotoxic T cell response. Whilst PD‐L1 antibodies act mainly at the TME, anti‐CTLA‐4 antibodies play a role in the local tumour draining lymph node. CTLA‐4 is expressed on the T cell surface in response to T cell receptor engagement and costimulatory signalling through CD28. The mechanism underlying the immune inhibitory function of CTLA‐4 inhibitors relies mainly on the competition of CTLA‐4 and CD28 in binding to the same ligands―CD80 and CD86. Abbreviations: APC ‐ antigen presenting cell, TME ‐ tumour microenvironment, MHC ‐ major histocompatibility complex, PD ‐ programmed death, CTLA ‐ cytotoxic T lymphocyte associated protein, CD ‐ cluster of differentiation, Treg ‐ T regulatory, TCR ‐ T cell receptor, BCR - B cell receptor.

Identifying patients most likely to benefit from these agents has become an increasingly important area of investigation. The current known biomarkers of response to ICI are illustrated in Figure 1. The expression of PD-L1 on tumour cells and PD-1 on immune cells have both been investigated as predictive biomarkers for anti-PD-1 therapies in clinical trials (Garon et al., 2015; Carbone et al., 2017; Schmid et al., 2020) and are currently used as biomarkers in NSCLC and urothelial cancers. Since a proportion of PD-L1 positive tumours do not respond to ICIs, and some PD-L1 negative tumours do respond, this suggests the response is complex and heterogenous. It has been suggested that both PD-L1 positivity and infiltration of CD8^+^ T cells are required for response (Ribas and Wolchok, 2018). In melanoma and NSCLC, response rates to anti-PD-1 treatment correlate with the presence of existing tumour infiltrating lymphocytes (TILs) and proliferation of cytotoxic CD8^+^ T cells at the tumour margin and intratumourally (Tumeh et al., 2014) (Maibach et al., 2020). In contrast the presence of immunosuppressive T regulatory (Treg) cells and myeloid derived suppressor cells (MDSCs) in the TME, dampen an immune response and carry a poor prognosis for patients (Umansky and Sevko, 2013; Marvel and Gabrilovich, 2015; Kotsakis et al., 2016; Weber et al., 2018). Furthermore, tumour intrinsic factors such as tumour mutational burden (TMB) (Hugo et al., 2016) and mismatch repair (MMR) deficiency causing microsatellite instability (MSI) (Le et al., 2015) are associated with response to ICI. Specific genetic mutations in immune pathways, such as PD-1 and PD-L2 ligand gene chromosome 9p24.1 amplification (Green et al., 2010) and the patient’s HLA genotype encoding their MHC class 1 molecules, can determine the efficacy of the anti-tumour immune response following ICI therapy (Goodman and et al., 2020). Despite these numerous different biomarkers, none can be used to consistently predict response or as markers to reliably select ICI responsive patients across all tumour types.

The anti-tumour immune response is complex and involves a number of different immune cell types which interact with tumour cells and with each other, starting within the TME and later within lymphoid structures. It is important to point out that it is believed the first step in a tumour immune response occurs within the primary tumour itself and is recognition of tumour cells as foreign. TMB, which has been predictive of ICI efficacy in some tumour types, gives rise to ‘foreign’ neoantigen production. Neoantigens expressed on the tumour cell surface or released when a tumour cell dies, are taken up by antigen presenting cells (APCs) in the TME. There are a number of cell types which are capable of presenting antigen, including tumour associated macrophages (TAMs), dendritic cells (DCs), monocytes and monocyte derived cells in the TME. These immune cells will next travel out of the primary tumour and into the tumour draining lymph node (TDLN), where tumour antigen has been found in abundance (Marzo et al., 1999). The immunogenic tumour antigens are presented to naïve T cells and the decision for tumour tolerance or immune activation will be made. In addition to the interaction of tumour and T cells, other cell types are instrumental in the anti-tumour immune response. Challenging the view that ICI activity occurs primarily at the tumour site, there is an increasing body of literature suggesting the importance of other cell types outside of the TME and in TDLNs (Fransen et al., 2018; Dammeijer et al., 2020; Meyers and Banerji, 2020).

Research focusing on the role of TDLN (used interchangeably with regional lymph nodes) in ICI therapy is limited but there is increasing evidence that they play a crucial role in mediating an anti-tumour response. A study of sentinel and axillary nodes of breast cancer patients demonstrated that immune cell populations, specifically CD4^+^ T cells and DCs, correlated with disease free survival, independent of lymph node metastasis (Kohrt et al., 2005). The immune profile within TDLNs is therefore of clinical importance. Indeed, in TDLN with metastasis there are increased numbers of T regulatory cells (Tregs) which express immune suppressive co-receptors and traffic to the TME (Nunez et al., 2020). The receptors expressed on such cells are targets for ICI therapy and therefore information from the TDLN may predict response. This review will cover the emerging literature on the potential for assessment of immunological responses in TDLN as predictive biomarkers for treatment with ICIs. Table 1 summarises the principal evidence that will be discussed below.

**TABLE 1:** Summarising the studies investigating the importance of TDLN in anti-tumour immunity following immunotherapy.

Pre-clinical studies	Study	Model	Conclusions
Evidence of Antigen presentation in TDLN	Chamoto et al. (2006)	Th-1 cell therapy in an OVA-expressing tumour murine model.	APC travel to TDLN presenting tumour antigen, resulting in tumour specific Th1 cell proliferation, immune infiltration and tumour regression.
	Marzo et al. (1999)	HA specific CD8^+^ T cell adoptive transfer in a murine HA-expressing tumour model.	Tumour antigen stimulates expansion of T cells in TDLN throughout tumour growth.
	Hargadon et al. (2006)	Evaluation of antigen presentation and CD8^+^ T cell function in an OVA-expressing melanoma tumour murine model.	Tumour derived antigen can be cross-presented by APCs or directly presented by tumour cells to naïve T cells in TDLN, and this induces CD8^+^ T cell differentiation.
	Dammeijer et al. (2020)	Assessment of TDLN in murine tumour (mesothelioma, melanoma, pancreatic and colon adenocarcinoma) models following TDLN-targeted PD-L1 blockade, correlated with PD-1/PD-L1 interactions in TDLN of non-metastatic melanoma patients (see below).	TDLNs contain tumour specific PD-1^+^ T cells co-localising with PD-L1 expressing myeloid cells, including cDCs. Selective targeting of PD-L1 TDLN-resident T cells affects a systemic anti-tumour immune response and tumour control.
	Salmon et al. (2016)	Assessment of immune cell subsets in a murine melanoma model following PD-L1 blockade.	CD103^+^ DCs, presenting tumour antigen in the TDLN were able to promote tumour specific antigen mediated T cell activation and proliferation. Expansion of CD103+ DCs following poly I:C administration enhanced tumour response to BRAF or PD-L1 blockade.
Tumour specific T cell activation and proliferation	Fransen et al. (2018)	Murine models of colon adenocarcinoma treated with anti-PD-1 and PD-L1 therapy.	TDLNs, but not non-draining LNs, contained CD11b^+^ myeloid cells with higher levels of PD-L1 expression and increased numbers and activation of CD8^+^ T cells after anti-PD-1 therapy. Surgical resection of TDLN and inhibition of lymphocyte trafficking from LN abolished therapy induced tumour responses.
	Principe et al. (2020)	Murine tumour models (mesothelioma, renal cell carcinoma) were treated with anti-CTLA-4 and anti-PD-L1 therapy then tumours and TDLN assessed for immune cell infiltration and profiling.	ICI therapy increased the proliferation of antigen-specific cytotoxic T cells with an effector memory phenotype both in the tumour and TDLN, and correlated with ICI response.
	Chamoto et al. (2017)	Murine tumour models (colon adenocarcinoma, lung carcinoma and fibrosarcoma) treated with anti-PD-L1 and targeted therapies.	Resection of TDLNs prior to tumour implantation and/or prior to anti-PD-1 treatment completely abolished or reduced efficacy respectively. CTLs in TDLNs express other targets (e.g. MTOR) which can be drugged to provide synergistic responses with anti-PD-1 therapy.
	Liu et al. (2016)	Murine models of breast cancer treated with neoadjuvant or adjuvant immunotherapy.	Neoadjuvant immunotherapy gave greater therapeutic efficacy compared with adjuvant treatment, and correlated with sustained peripheral anti-tumour immune responses.
Migration of immune cell populations from TDLN to the TME	Spitzer et al. (2017)	Murine breast cancer model treated with anti-PD-1 therapy and other immunotherapies. Immune cell responses were analysed from multiple tissues using mass cytometry. Results were correlated with melanoma patients responding to immunotherapy.	T reg, CD8^+^ T cells and CD4^+^ effector memory T cells had significantly increased proliferation demonstrating the initiation of a T cell-mediated immune response within the TDLN. In addition, memory T cells were able to activate naïve T cells in the TDLN for a sustained immune response. This CD4^+^ T cell subset was also found in the peripheral blood of patients with melanoma responding to anti-CTLA-4 + GM-CSF therapy.
	Fransen et al. (2018)	Murine models of colon adenocarcinoma treated with anti-PD-1 and PD-L1 therapy.	The inhibition of trafficking of T cells from the TDLN decreased T cell numbers in peripheral circulation and correlated with reduced efficacy of anti-PD-1 treatment. In addition, when TDLN were removed prior to therapy with anti-PD-1, the number of CD8^+^ T cells in the TME was reduced.
Immune tolerance in the TDLN	Watanabe et al. (2008)	Murine fibrosarcoma model receiving adoptive cell transfer with MDSCs from tumour bearing mice or normal splenocytes and stimulated with inoculation of tumour cells.	Immunosuppressive MDSCs have been isolated in TDLN and shown to dampen anti-tumour T cell responses, reducing T cell activation and CD4+/CD8+ T cell numbers but not T cell effector function.
	Huang et al. (2017)	Assessment of TDLN in a murine breast carcinoma model.	TGF-beta secreting Tregs within TDLNs suppress tumour specific CD8^+^ T cell cytotoxic activity, resulting in tumour growth. Surgical resection of TDLN reduced distant metastasis.
	Alonso et al., (2018)	Assessment of TDLN in a murine lung adenocarcinoma model.	In early tumour development, CD4^+^ T cells are driven to differentiate as Tregs rather that effector CD4^+^ T cells in the TDLN, promoting suppressive Treg responses that mimic peripheral self-tolerance to tumour antigen.
Clinical studies	Study	Tumour type	Conclusions
	Kohrt et al. (2005)	Assessment of sentinel and axillary lymph nodes in breast cancer patients.	Presence of CD4^+^ T cells and DCs in TDLN correlate with disease free survival.
	Nunez et al. (2020)	Assessment of tumour invaded and non-invaded TDLNs in breast cancer patients.	T reg cells traffic from cancer TDLN to the TME.
	Sluijter et al. (2015)	Melanoma tumour and sentinel lymph node assessment following randomised trial of intradermal CpG-B/GM-CSF.	The human equivalent CD141^+^ CLEC-9A^+^ DCs have been found in TDLNs and were responsible for the cross presentation and activation of anti-tumour T cells in melanoma patients.
	Dammeijer et al. (2020)	PD-1/PD-L1 interactions in TDLN of non-metastatic (stage II) melanoma patients, correlated with findings in TDLN in murine tumour models following TDLN-targeted PD-L1 blockade (see above).	PD-1/PD-L1-interactions in TDLNs of non-metastatic melanoma patients, but not in the corresponding primary tumours, are associated with early distant disease recurrence.
	Kamphorst et al. (2017)	Assessment of peripheral blood from NSCLC patients following anti-PD-1 therapy, correlated with *in vivo* findings following anti-PD-1 therapy in a murine model of colon carcinoma.	CD28/B7 mediated proliferation of CD8^+^ T cells is required for anti-tumour efficacy and occurs in TDLNs.
	van de Ven et al. (2017)	Assessment of endobronchial fine needle aspirates from TDLN in NSCLC patients.	TDLN has a greater number of tumour-antigen experienced immune cells and specific activated T cell subsets than peripheral blood sampling.
	Shariati et al. (2020)	Assessment of TDLN in breast cancer patients.	CD86^+^ B cells in TDLN were associated with higher tumour grade and a greater number of metastatic lymph nodes. Expression of PD-1 and CD39 on B cells in LNs correlated with higher grade and larger tumours respectively. Patients with CD73^+^ B cells had fewer involved lymph nodes.
	DeFalco et al. (2018)	Assessment of peripheral blood samples from metastatic melanoma, renal cell carcinoma and lung adenocarcinoma patients.	High levels of blood plasmablasts were found in patients with stable metastatic disease, suggesting that B cell responses may be important for tumour control.

## Lymph Node Assessment for Immune Response to Immune Checkpoint Inhibition

### Antigen Presentation

One of the most common measures of immune response is the degree of antigen presentation which can occur in numerous different areas throughout the body. It is important to try and characterize the order of which locations antigen presentation occurs and which cell types are involved in an anti-tumour response, a phenomenon that is still under investigation. Chamoto et al. compared anti-tumour immune responses in TDLN, distal LN and the spleen of mice by measuring the production of tumour-specific cytotoxic lymphocytes (CTLs). They demonstrated that APCs which had taken up fluorescently labelled ovalbumin antigen (OVA) tumour antigen travelled first to TDLN, and that migration of TAMs was increased following systemic injection of tumour-specific Th1 cells (Chamoto et al., 2006). These Th1 cells proliferated in TDLN after exposure to DCs carrying tumour antigen, and to a greater extent than in other examined lymphoid tissue sites. In addition, there was an increase in tumour specific CD8^+^ T cells in TDLN and CD8^+^ TILs which correlated with tumour regression. This was consistent with earlier work (Marzo et al., 1999), which demonstrated that tumour antigen can stimulate clonal expansion of tumour specific T cells in the TDLN but not in distant/non-draining lymph nodes or within the TME. Together, this work highlights the importance of the TDLN as a site responsible for the initiation of the anti-tumour immune response.

Since the activation of T cells for an immune response relies upon the presentation of antigen from the tumour, research has focused on identifying the specific cell types involved in antigen presentation in the TDLN since this is presumed to be the first place formal antigen presentation occurs. Hargadon et al. demonstrated that murine melanoma tumour derived antigen can be cross-presented by APCs or directly presented by tumour cells to naïve T cells in TDLN, and that this induces CD8^+^ T cell differentiation (Hargadon et al., 2006). Whilst cross-presentation remained effective in late stage tumour growth, direct presentation occurring in TDLN containing metastases resulted in incomplete CD8^+^ T cell differentiation which was likely due to tumour mediated immunosuppressive changes to APCs and the TME. Professional APCs such as conventional dendritic cells (cDCs) express PD-L1 and migrate to the TDLN. Dammeijer et al. demonstrated in murine models of mesothelioma, melanoma and pancreatic adenocarcinoma that TDLNs contain tumour specific PD-1^+^ T cells co-localising with PD-L1 expressing myeloid cells, including CD11c^+^ conventional dendritic cells (cDCs) (Dammeijer et al., 2020). By selectively targeting PD-L1 only in the TDLN, they demonstrated that TDLN-resident T cells are able to affect a systemic anti-tumour immune response to control the distant tumour site. This suggests that cDCs play a significant role in initiating T cell responses in the TDLN.

Furthermore, work by Salmon and colleagues identified the CD103^+^ population of DCs as the APCs responsible for tumour antigen presentation in the TDLN in a murine model of melanoma (Salmon et al., 2016). This tissue resident subtype of DCs is known to cross present antigen to CD8^+^ T cells and is involved in cell-priming. Indeed, whilst macrophages and monocytes were abundant in the tumour, tumour-infiltrating DCs were sparse. Fluorescently labelled tumour antigen was taken up most by TAMs but transport to the TDLN was achieved only by the CD103^+^ DC subpopulation. Furthermore, when LN resident and migratory DCs were isolated from the TDLN of mice with melanoma tumours expressing OVA, migratory CD103^+^ DCs were uniquely able to promote tumour ova-specific antigen mediated T cell activation and proliferation (Salmon et al., 2016). Identifying this subtype of APC as significant mediators of the anti-tumour immune response led to evaluation of their role in ICI treatment efficacy in the same model. Interestingly, PD-L1 was more highly expressed on CD103^+^ DCs in the TDLN than in non-TDLN. Anti-PD-L1 antibody treatment delays melanoma tumour growth in this model but, despite PD-L1 expression in tumour cells, the anti-tumour effect of PD-L1 blockade was reduced in *Batf3−/−* mice which lack CD103^+^ DCs (Salmon et al., 2016). In addition, strategies to increase expansion of CD103^+^ DCs expressing PD-L1 also increased ICI efficacy for tumour regression and correlated with increased CD8^+^ T cells in TDLN and tumours. The human equivalent CD141^+^ CLEC-9A^+^ DCs have been found in TDLNs and were responsible for the cross presentation and activation of anti-tumour T cells in melanoma patients (Sluijter et al., 2015). Identifying these DC subsets and their expression of CD141, CLEC-9A and PD-1 in patients may have predictive role in ICI therapy. Overall, preclinical models provide evidence that antigen presentation by different subtypes of DCs within TDLN is an important first step in immune activation. Translation of this work in samples from patients with different tumour types is necessary to fully recognize the implications of DCs in the anti-tumour response, and more specifically in the anti-tumour response following treatment with ICIs.

### T Cell Activation and Proliferation for Anti-Tumour Cytotoxicity

T cell activation and proliferation is one of the most important outcomes of antigen presentation and initiates tumour cell elimination. Both activation and proliferation can occur within the TME or within a TDLN. This is also one of the best studied read-outs of successful immune activation by immunotherapy including ICI. It is understood that PD-1 inhibits the co-stimulatory receptor CD-28 and B7 ligand signalling which is responsible for T cell co-stimulation. Kamphorst et al. used murine colorectal and melanoma cancer models to demonstrate that CD-28 mediated signalling was responsible for anti-tumour CD8^+^ T cell responses (Kamphorst et al., 2017). The same group found that in patients with advanced NSCLC PD-1^+^ CD8^+^ T cells from peripheral blood samples, which were activated following anti-PD-1 therapy, were largely CD-28^+^. This suggests that CD-28 signalling is also important in the proliferation of cytotoxic T cells responsible for anti-tumour responses in patients. Since most tumours do not express B7 molecules, B7 expressing APCs are likely to play an important role in anti-PD-1 therapy efficacy. Furthermore, the majority of CD8^+^ TILs in NSCLC did not express CD-28 and therefore may be less responsive and less proliferative than T cells within TDLN following anti-PD-1 therapy (Kamphorst et al., 2017). Therefore, the authors suggest that proliferation of CD8^+^ T cells is required for anti-tumour efficacy and occurs in TDLNs. Therefore, expression of CD-28 by T cells may be an important biomarker for CD-28 mediated signalling in TDLNs and peripheral immune cells, and consequently for efficacy of anti-PD-1/PD-L1 ICI therapy.

A second study demonstrated that TDLN of NSCLC patients could be assessed by fine needle aspirate to determine the immune cell composition (van de Ven et al., 2017). They identified similar numbers of Tregs in TDLN and peripheral blood samples, but higher levels of PD-1 expression in CD8^+^ T cells and non-suppressive CD4^+^ T cells in TDLN vs. peripheral blood or non-draining LNs. In addition a PD-1 expressing activated CD4^+^ T cell population was present in TDLN but not peripherally. Together, these findings suggest that the TDLN has a greater number of tumour-antigen experienced immune cells and specific activated T cell subsets. Similarly another group demonstrated in an *in-vivo* model of colorectal cancer that TDLNs, but not non-draining LNs, contained CD11b^+^ myeloid cells with higher levels of PD-L1 expression and increased numbers and activation of CD8^+^ T cells after anti-PD-1 therapy (Fransen et al., 2018). The levels of PD-1 expression and presence of such immune cell populations may be able to predict response to PD-1/PD-L1 therapy.

The efficiency of T cell-mediated immunity relies upon a combination of cytokine production and signalling for differentiation and activation of T cell subsets required for cytotoxicity and immunological memory. These tumour-specific cytotoxic T cells are frequently immunologically exhausted after prolonged antigen exposure. T cell exhaustion is characterised by upregulation of the expression of inhibitory receptors including PD-1 and CTLA-4, which leads to reduced function and capacity for proliferation. Therefore, blockade of PD-1/PD-L1 and CTLA-4 improves T cell functioning. The level of exhaustion in T cells is directly influenced by their local microenvironment (Hope et al., 2019). Anti-tumour cytotoxic T cells in TDLNs are less exhausted compared with TILs, and therefore may play a greater part in the immune response (Hope et al., 2019). Evaluation of the presence of T cell subsets and markers of exhaustion and activation within TDLN may inform their contribution to tumour elimination by ICI.

Whilst exhausted T cells are often thought to have terminally differentiated, there is increasing evidence that in response to ICI treatment a subset of such T cells are able to differentiate (Tumeh et al., 2014; Siddiqui et al., 2019; Principe et al., 2020). In a murine tumour model ICI therapy increased the proliferation of antigen-specific cytotoxic T cells with an effector memory phenotype both in the tumour and TDLN (Principe et al., 2020). Tumours with greater infiltration of such effector memory T cells, and lower frequencies of T regulatory cells, responded better to ICI therapy. Understanding the profile of T cell subsets present in the TME and TDLN may be informative to predict ICI responses.

The importance of TDLN mediated immune responses has also been demonstrated in preclinical models using ICIs that are widely used in the clinic. In murine models testing PD-1 blockade, the efficacy of treatment was abolished by the ablation of TDLN or depletion of CD8^+^ T cells (Chamoto et al., 2017). Furthermore, surgical resection of TDLNs prior to tumour implantation and/or prior to anti-PD-1 treatment, completely abolished or reduced efficacy respectively. These studies support the idea that TDLN are central for inducing CTL mediated anti-tumour cell killing following PD-1 blockade. This is in support of earlier data in murine models of metastatic breast cancer where neoadjuvant immunotherapy gave greater therapeutic efficacy compared with adjuvant treatment, and correlated with sustained peripheral anti-tumour immune responses (Liu et al., 2016).

These studies highlight the evidence that supports TDLN as the first and likely most important site for APC interaction with T cells to induce initial tumour-specific CTL expansion. Since it is this interaction between tumour and immune cells which many ICIs target, the microenvironment in the TDLN is likely to impact their efficacy. Measuring the presence or activation status of T cells specifically located within the TDLN could become an easier and more informative measure of therapy efficacy than the assessment of TILs within the primary TME.

### Trafficking to Tumour Microenvironment and Other Associated Changes

The presence of particular T cell subsets, such as CD8^+^ T cells, infiltrating the tumour has been associated with ICI responses (Tumeh et al., 2014). Preclinical studies support the hypothesis that T cells originate in sites distant to primary tumour, such as the TDLN, and migrate to the primary tumour following initiation of therapy with ICI (Spitzer et al., 2017). Using a murine model of triple negative breast cancer, Spitzer et al. analysed the immune cell populations in the TME and TDLN following treatment with a combination of allogenic tumour binding IgG, anti-CD40 antibody and interferon-γ (Spitzer et al., 2017). They observed activation of macrophages and increases in B cell subsets, innate immune cells and clusters of intra-tumoural CD8^+^ and memory CD4^+^ T cells and Tregs three days following therapy. However, there were no changes in proliferation rate of these immune cell subsets eight days after treatment, when the tumour regressed, suggesting that other lymphoid cells may be the source of immune cells which drive tumour regression. Indeed, in the TDLN there was expansion of activated and naïve B cells, NK cells, DCs and Th1 T cells. T reg, CD8^+^ T cells and CD4^+^ effector memory T cells also had significantly increased proliferation demonstrating the initiation of a T cell-mediated immune response within the TDLN (Spitzer et al., 2017). During tumour regression there was also proliferation in naïve T cell subsets in the TDLN, suggesting that memory T cells were able to activate naïve T cells for a sustained immune response. Therefore, immunological activity from the TDLN plays a greater role toward the efficacy of the anti-tumour immune response than the TME. Further analysis of the T cells subsets pointed specifically to CD4^+^ T cells effecting the tumour immune response. *In vivo* transfer of peripheral CD4^+^ T cells provided a more durable tumour regression compared with peripheral CD8^+^ T cells, in contrast to previous studies (Chen and Mellman, 2013). The group also identified elevated numbers of this same CD4^+^ T cell subset in melanoma patients responding to anti-CTLA-4 antibody treatment, compared with non-responders.

Anti-tumour T cell cytotoxicity is also dependent on efficient trafficking and infiltration at the tumour site. In one study T cells that had been activated in the TDLN were prevented from traveling to the tumour site using a S1P receptor inhibitor FTY720, trapping T cells in lymphoid tissue. Inhibition of FTY720 led to decreased T cell numbers in peripheral circulation and correlated with reduced efficacy of anti-PD-1 treatment. In addition, when TDLN were removed prior to therapy with anti-PD-1, the number of CD8^+^ T cells in the TME was reduced (Fransen et al., 2018). These data support the notion that T cells from outside the TME, in this case from TDLN, play an important role in ICI responses.

### Immune Tolerance in the Tumour Draining Lymph Node

Immune evasion is a hallmark of cancer and so the immune microenvironment in TDLNs often supports immunological tolerance. Therefore, it is also an important site to identify potential negative biomarkers for ICI response. TDLNs are understood to be a site of Treg activation and the generation of new tumour-specific regulatory T cells (Tregs) (Munn and Mellor, 2006). Therefore, they develop a certain level of immune privilege, similar to that which is observed in the TME and which allows tumours to grow despite high levels of circulating tumour-specific cells (Rosenberg et al., 2005). To a lesser extent than the TME, immunosuppressive MDSCs have also been isolated in TDLN (Watanabe et al., 2008) and can dampen T cell responses. It is likely that the microenvironment in the TDLN is dynamic and immune reactivity to tumour antigen is altered not only by the presence of immunosuppressive MDSCs but also by the presence of metastatic tumours. Furthermore, TGF-beta secreting Tregs have also been identified within TDLNs, and are able to suppress tumour specific CD8^+^ T cells cytotoxic activity resulting in tumour growth in breast cancer models (Huang et al., 2017). Alonso et al. suggest that, even in early tumour murine lung adenocarcinoma development, CD4^+^ T cells are driven to differentiate as Tregs rather that effector CD4^+^ T cells in the TDLN (Alonso et al., 2018). They suggest that the anti-tumour CD4^+^ T cell response is inhibited and the suppressive Treg response is promoted in the TDLN, mimicking peripheral self-tolerance to tumour antigen. Overall, these studies suggest that the presence of primary and metastatic disease promote immune suppression within the TDLN and this may need to be overcome to observe a response to ICI.

### B Cell Antibody Responses

The presence and activity of antigen presenting cells and T cells of particular subsets in the TDLN may be useful to predict outcome of immunotherapy. In addition, the presence of B cells and existence of tertiary lymphoid structures may also be informative.

Tumour infiltrating B cells are capable of binding tumour proteins and processing them to antigens for presentation to T cells. Once activated, B cells can release antibodies to bind to tumour cells to trigger antibody-dependent cell death. In breast cancer the number and subset of B cells present in patient lymph nodes has been correlated with prognosis. CD86^+^ B cells were associated with higher tumour grade and a greater number of metastatic lymph nodes. Furthermore, the expression of PD-1 and CD39 on B cells in LNs correlated with higher grade and larger tumours respectively (Shariati et al., 2020). In contrast, patients with B cells expressing CD73 had fewer involved lymph nodes. Further research is needed to determine the role of such B cell subsets in the anti-tumour immune response and in the TDLN. In another study, patients with stable metastatic melanoma, renal cell carcinoma and lung adenocarcinoma had high levels of blood plasmablasts suggesting that B cell responses may be important for tumour control (DeFalco et al., 2018). The authors demonstrate B cell antibody responses are to ‘public’ tumour antigens, rather than patient-specific, and that these antibodies could direct tumour killing *in vivo*. B cell responses may therefore be of therapeutic and predictive value to improve ICB therapy. Indeed, B cell infiltration and the presence of lymphoid structures (TLS) in patient tumours have been associated with response to ICB (Cabrita et al., 2020; Helmink et al., 2020; Petitprez et al., 2020). Importantly, the presence of tumour TLS prior to therapy may be predictive for a response to ICB (Petitprez et al., 2020) which could enable better patient selection.

## Conclusion

This review has discussed compelling evidence that TDLNs are an important site for anti-tumour immune responses. The presence, activation status and infiltration of particular immune cells, namely T cell subsets from the TDLN to the tumour have been associated with ICI responses. Identifying the presence and status of antigen presenting cells, T cell subsets and B cells in the TDLN may be useful to select those patients who have an abundance of immune suppressed cells expressing checkpoint receptors targeted by ICI therapy. At present predictive biomarker studies frequently focus on material from the tumour itself or cells in circulation taken from serum samples and do not take in to account the immune status of the TDLN prior to, or during therapy. It will be important to include pre and post treatment lymph node sampling in ICI clinical trials to understand the predictive value of these findings.

Furthermore, *in vivo* work has demonstrated that the resection of TDLN prior to ICI therapy abolished tumour response and reduced immune cell infiltrate in the TME (Fransen et al., 2018). This raises a clinical consideration for the timing of therapy with ICI. It is possible that adjuvant ICI therapy following removal of sentinel or TDLN groups may significantly alter response. There may be an argument for neoadjuvant ICI therapy, whilst TDLN remain *in situ* rather than adjuvant ICI therapy, to maximise the interaction between TME and TDLN immune cell populations.

Indeed, if TDLN remain *in situ* prior to ICI, pre-therapy biopsy of TDLN or sampling of systemic immune populations may prove important predictive biomarkers. Post therapy evaluation of resected TDLN may be informative for prognosis or to determine the need for further adjuvant therapy. Ongoing clinical trials of ICI in the neoadjuvant setting may prove informative to determine the most efficacious timing for therapy. TDLN may be essential for the efficacy of ICI and a rich site to identify new predictive biomarkers.
